# Irisin Promotes Human Umbilical Vein Endothelial Cell Proliferation through the ERK Signaling Pathway and Partly Suppresses High Glucose-Induced Apoptosis

**DOI:** 10.1371/journal.pone.0110273

**Published:** 2014-10-22

**Authors:** Haibo Song, Fei Wu, Yuan Zhang, Yuzhu Zhang, Fang Wang, Miao Jiang, Zhongde Wang, Mingxiang Zhang, Shiwu Li, Lijun Yang, Xing Li Wang, Taixing Cui, Dongqi Tang

**Affiliations:** 1 Center for Stem Cell & Regenerative Medicine, The Second Hospital of Shandong University, Jinan, P.R.China; 2 Shandong University Qilu Hospital Research Center for Cell Therapy, Key Laboratory of Cardiovascular Remodeling and Function Research, Qilu Hospital of Shandong University, Jinan, P.R.China; 3 Department of Cell Biology and Anatomy, University of South Carolina, Columbia, South Carolina, United States of America; 4 Center for Reproductive Medicine, Zibo Maternal and Child health hospital, Zibo, P.R.China; University of Hull, United Kingdom

## Abstract

Irisin is a newly discovered myokine that links exercise with metabolic homeostasis. It is involved in modest weight loss and improves glucose intolerance. However, the direct effects and mechanisms of irisin on vascular endothelial cells (ECs) are not fully understood. In the current study, we demonstrated that irisin promoted Human Umbilical Vein Endothelial Cell (HUVEC) proliferation. It was further demonstrated that this pro-proliferation effect was mediated by irisin-induced activation of extracellular signal–related kinase (ERK) signaling pathways. Inhibition of ERK signaling with U0126 decreased the pro-proliferation effect of irisin on HUVECs. It was also demonstrated that irisin reduced high glucose-induced apoptosis by up-regulating Bcl-2 expression and down-regulating Bax, Caspase-9 and Caspase-3 expression. In summary, these results suggested that irisin plays a novel role in sustaining endothelial homeostasis by promoting HUVEC proliferation via the ERK signaling pathway and protects the cell from high glucose-induced apoptosis by regulating Bcl-2,Bax and Caspase expression.

## Introduction

Many vascular diseases are caused by endothelial cell (EC) injury and dysfunction, which occurs in chronic metabolic diseases such as metabolic syndrome and type II diabetes mellitus [1], [2]. In many chronic metabolic diseases, vascular endothelial integrity is affected by EC proliferation and apoptosis, which assures blood vessel function [3]. Therefore, restoration of injured EC via regulating endothelial cell proliferation and apoptosis may have very important significance. Thus, extensive efforts were made to find more metabolic related factors that can promote endothelial cell proliferation and avoid their death, but the outcomes were not encouraging.

The benefits of exercise in metabolic and cardiovascular disease prevention and progression have been well documented [4]. Irisin is a newly discovered myokine that links exercise with increased energy expenditure to produce fundamental exercise-based health benefits [5]. Irisin is released from skeletal muscles and is increased with exercise when the fibronectin type III domain containing 5 (Fndc5) is proteolyzed. Irisin is highly conserved across species [5]. Irisin has been proposed to be a bridge between exercise and metabolic homeostasis and to be involved in modest weight loss and improved glucose intolerance in mice [5]. Recent studies discovered that type 2 diabetic patients displayed significantly lower levels of circulating irisin compared with non-diabetic control subjects [6], [7]. Circulating irisin levels were decreased in patients with chronic kidney disease (CKD) and were independently associated with high-density lipoprotein cholesterol levels [8]. Intriguingly, a new study demonstrated that pharmacological irisin concentrations promote mouse H19-7 HN cell proliferation via the STAT3 signaling pathway [9]. This finding suggests that irisin may have a pro-proliferation effect in addition to its role in regulating metabolic homeostasis. However, no previous studies have evaluated whether irisin may directly regulate human EC.

In this study, we treated Human Umbilical Vein Endothelial Cells (HUVECs) with human recombinant irisin (r-irisin), which was expressed and purified in our laboratory to detect its direct effects on HUVEC proliferation and apoptosis [10]. The possible signaling mechanisms by which irisin exerts its effects were also characterized. These studies demonstrated for the first time that irisin can promote HUVEC proliferation via extracellular signal–related kinase (ERK) pathway activation. Irisin can also reduce high glucose-induced apoptosis by up-regulating Bcl-2 and down-regulating Bax, Caspase-9 and Caspase-3 expression.

## Materials and Methods

### Expression and Purification of Human Irisin

The expression and purification of human irisin were performed as previously described [10]. Briefly, the cDNA (360 bp) of human irisin was designed and synthesized by Life Technologies. The synthesized human irisin cDNA was cloned into EcoR1/Xba1 sites of the pPICZaA plasmid. A linearized pPICZaA-irisin plasmid was use to transforme the P. pastorisX-33 according to the kit manual (PichiaEasycomp Transformation Kit; Invitrogen). The induction of protein expression and culture of yeast were performed as previously described [10]. Then the r-irisin protein in the supernatant was purified and used in our study.

### Primary culture of Human Umbilical Vein Endothelial Cells (HUVECs)

Human umbilical vein endothelial cells(HUVECs) were isolated from human umbilical cords using 300 units/ml collagenaseII(Sigma-Aldrich, St. Louis, MO) and cultured in Medium 199 (Invitrogen) with 10% (v/v) fetal bovine serum (FBS, Invitrogen) and conditioned supplement (10 ng/mL EGF and 10 ng/mL bFGF,Peprotech) at 37°C in a 5% CO_2_ and 95% air atmosphere. HUVECs were used at passages 3–6 in all of the experiments.

The study protocol conformed to the ethical guidelines of the 1975 Declaration of Helsinki with the approval of the Institutional Medical Ethics Committee of Qilu Hospital, Shandong University. All of the donors provided written informed consent.

### [3H] Thymidine uptake

HUVECs (P6) were cultured in M199 medium with 10% FBS along with EGF(10 ng/mL, Peprotech) and bFGF(10 ng/mL, Peprotech) at 50% confluence. The cells were cultured in serum-free M199 for 24 hours and treated with or without irisin for 40 hours. [3H] thymidine (final concentration 1 uCi/ml) was added to the media during the last 24 hours of culture. After washing with ice-cold PBS two times, the cells were precipitated with ice-cold 5% trichloroacetic acid (TCA, Sigma-Aldrich, St. Louis, MO) for 4 hours and washed with ice-cold 5% TCA two times followed by two additional washes with ice-cold PBS. Then, they were lysed with 0.2 ml 0.5 M NaOH for 30 minutes at 37°C. DNA synthesis was measured by [3H] thymidine uptake.

### Cell Counting

HUVECs were planted in a 10-cm diameter culture dish in the presence or absence of 20 nM Irisin. After digestion with trypsin every 24 h, the cells were subjected to cell size calculation using a counting slide (BIO-RAD) according to the manufacturer's instructions. The data were analyzed to investigate the influence of irisin on HUVEC proliferation. All of the experiments were performed in triplicate.

### Immunofluorescent staining

HUVECs were grown on glass cover slips and fixed with 4% paraformaldehyde and permeabilized with 0.5% Triton X-100. Then, the cells were placed in 5% normal goat serum for 1 hour. They were then incubated with rabbit anti-Ki67 (1∶500, Cat. No. ab15580, Abcam) antibodies overnight at 4°C. The cells were visualized with Alexa Fluor 640 conjugated goat anti-rabbit IgG (Invitrogen) for 1 hour at room temperature. The cells were incubated in 4′, 6-diamidino -2-phenylindole (DAPI)/PBS (1∶5000, cat. no. D9542, Sigma-Aldrich, St. Louis, MO) for 3 minutes at room temperature and washed 3 times in PBS for 5 minutes per wash. Finally, the cells were photographed using a Nikon eclipse Ti and UltraVIEW:emoji:VOX confo- cal microscope, and images were analyzed using Volocity software (PerkinElmer).

### Western blot

Total cell protein concentrations were determined using the BCA protein assay kit (Pierce, Rockford, IL, USA). Equal amounts of protein from cell lysates were loaded in 12% sodium dodecyl sulfate-polyacrylamide gels. After electrophoresis, proteins were transferred to polyvinylidene fluoride membranes, blocked with 5% fat-free milk at room temperature for 1 h, and incubated with the indicated primary antibodies overnight at 4°C at 1∶1000 dilution (rabbit anti-ERK1/2, anti-phospho-ERK1/2, anti-Akt, anti-phospho-Akt, anti-P38 MAPK, anti-phospho-P38 MAPK anti-phospho-GSK-3β, anti-GSK-3β,, anti-Bcl-2, anti-Bax, anti-Bad, anti-Caspase-9, anti-Caspase-3 antibodies [Cell Signaling Technology, Inc.]). Then, the membranes were washed for 15 min three times with tris-buffered saline with Tween 20 and incubated with HRP-conjugated secondary antibodies for 1 h at room temperature. After washing again for 15 min three times with Tris-buffered saline with Tween 20, immune complexes were detected with enhanced chemiluminescence reagents, and the blots were quantified by densitometric analysis using the Alpha Imager 2200.

### Apoptosis Analysis

HUVEC apoptosis was determined with the annexin V-FITC/propidium iodide assay. HUVECs were seeded into 6-well plates at 1×10^6^/well in M199 containing 10% FBS, 10 ng/ml bFGF and 10 ng/ml EGF. After starvation in serum free media for 24 h, the cells were treated with or without 20 nM Irisin and 30 mM glucose for 24 h. They were then harvested, washed and re-suspended in PBS. Apoptotic cells were determined with an Annexin V-FITC apoptosis detection kit (BD Biosciences, USA) according to the manufacturer's protocol. Briefly, the cells were washed and subsequently incubated for 15 minutes at room temperature in the dark in 100 µl 1× binding buffer containing 5 µl Annexin V-FITC and 10 µl PI. Apoptosis data were determined by the BD accuriC6 flow cytometer and processed using FlowJo(FlowJo, Ashland, OR, USA) software.

### Statistics

The data are expressed as the mean±standard deviation (SD). All of the experiments were repeated at least three times. Comparisons among values for all groups were performed by one-way analysis of variance (ANOVA). Holm's t-test was used for analysis of differences between different groups. Differences were considered to be significant at P<0.05.

## Results

### Effect of irisin on HUVEC proliferation

**Figure 1:**
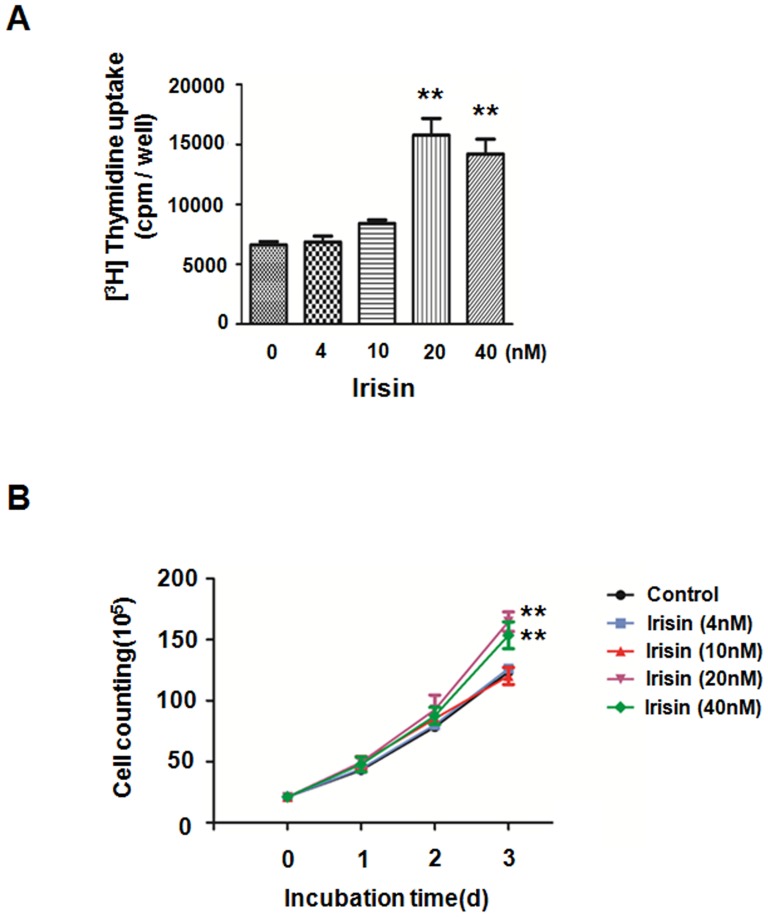
Irisin promoted HUVEC proliferation. (A) [^3^H] thymidine uptake in HUVECs that were cultured in M199 and treated with or without irisin at the indicated concentrations for 40 h. The data were expressed as the mean ± SE of three independent experiments,**p<0.01 vs. the untreated group. (B) Growth curves of HUVECs. HUVECs treated with or without irisin were constructed by plotting cell numbers that were counted using a hemocytometer over three days of incubation. ** p<0.01 vs. untreated, the data were expressed as the mean±SE of three independent experiments.

**Figure 2:**
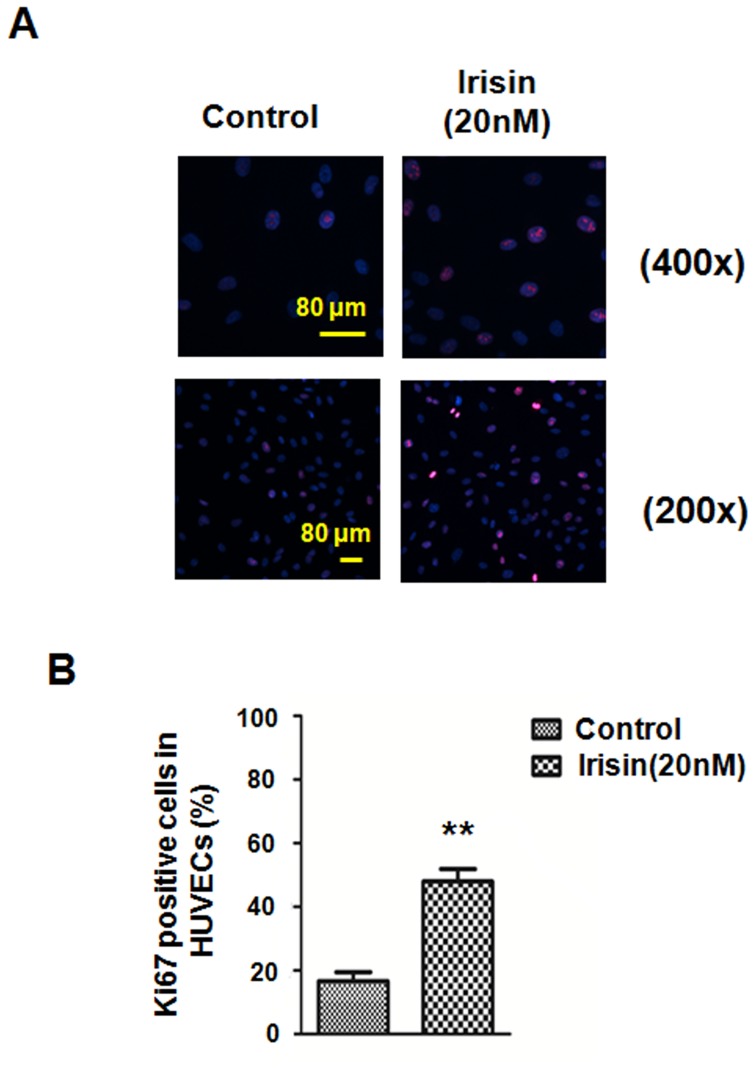
Ki67 staining in HUVECs. (A) Ki67 staining in HUVECs that had been treated with PBS (control) or irisin (20 nM) for 24 h. Images were taken with a confocal microscope. (B) Proliferation was assessed by the ratio of the average number of Ki67-positive cells to total cells in 10 random high-magnification fields. The data were expressed as the mean ± SE of three independent experiments,**p<0.01 vs. the untreated group.

To investigate the role of irisin on HUVEC proliferation, [^3^H] thymidine uptake was measured as described in the materials and methods. It was observed that the increase of [^3^H] thymidine uptake induced by irisin (20 nM) was 2.4 times higher than the control group in serum-free conditions (Fig. 1A). To assess the actual cell number changes, the effects of irisin on HUVEC proliferation was detected by direct cell counting. The result demonstrated that irisin over a range of concentrations (20 and 40 nM) can significantly accelerate HUVEC proliferation (Fig. 1B). Considering that the maximum effect appeared when the irisin concentration was 20 nM, 20 nM Irisin was chosen for the following experiments. To further reveal the effect of irisin on HUVEC proliferation, anti-Ki67 immunofluorescent staining was used. Ki67 is a nuclear protein which is expressed in proliferating cells; thus, it may be essential for maintaining cell proliferation [11]. The results demonstrated that there were more Ki67-expressing cells in the irisin stimulation group than the control group (48.0±4.0% and 16.6±2.9%, respectively) (Fig. 2A), and the difference was statistically significant (P<0.01) (Fig. 2B).These data taken together suggested that irisin effectively promoted HUVEC proliferation in serum-free medium.

### Irisin Mediates HUVEC Proliferation through the ERK Signaling Pathway

**Figure 3:**
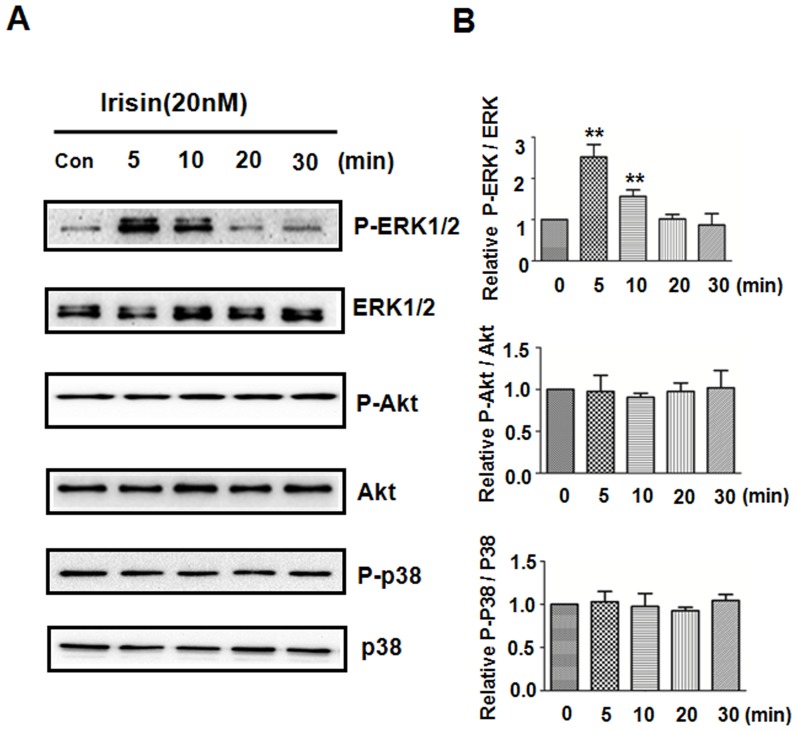
Irisin mediates proliferation via the ERK pathway. HUVECs were treated with or without irisin (20 nM) at indicated time points. (A) Phospho- and total ERK, p38 and AKT levels in cell lysates were analyzed by Western blot. (B) Densitometric analysis of the related bands was expressed as the relative optical band density, which was corrected using respective total proteins as a loading controls and normalized against the untreated control. The data were expressed as the mean ± SE of three independent experiments,**p<0.01 vs. untreated.

To gain further insights into the relationship between irisin and HUVEC proliferation, the protein levels of ERK, p38 MAPK, and AKT signaling molecules, which are critically involved in cell proliferation, were investigated. As demonstrated in Figure 3, after irisin treatment of HUVECs, phosphorylated ERK (P-ERK) levels were significantly increased at 5 minutes, peaked between 5 and 10 minutes, and decreased at 20 minutes (Fig. 3A). The increase was statistically significant, as quantified by densitometry (Fig. 3B). However, treating HUVECs with irisin had no effect on the level of phospho-p38 and AKT, indicating that the p38 and AKT signaling pathways are not involved in irisin-mediated cell proliferation (Fig. 3A and B).

**Figure 4:**
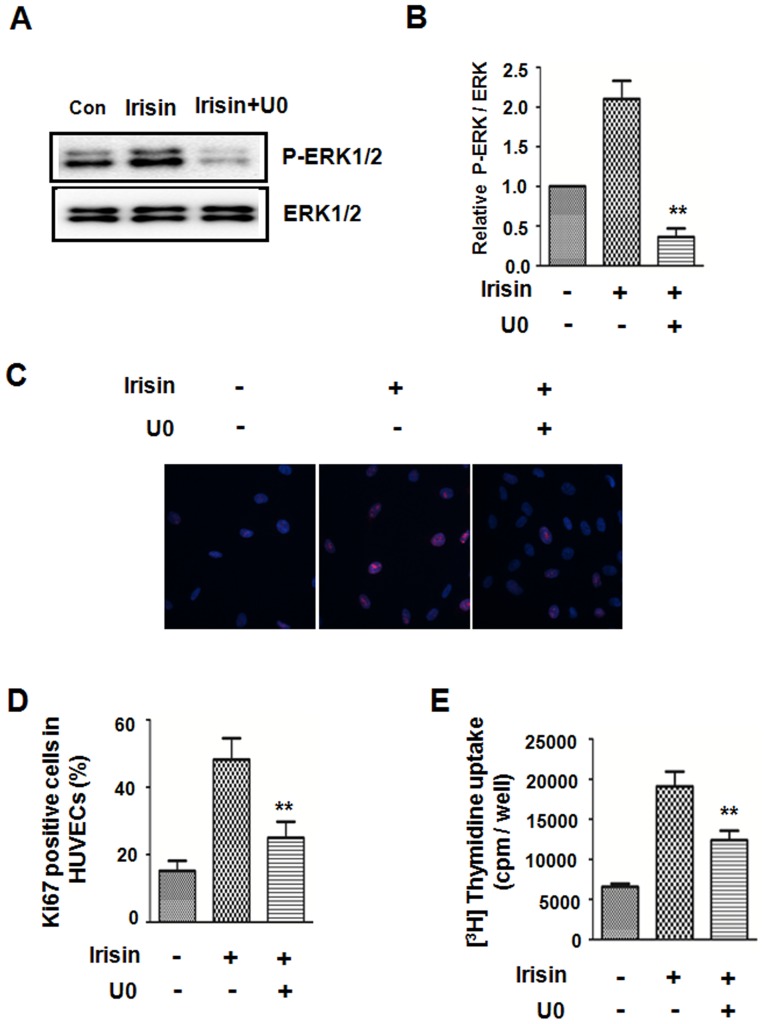
ERK inhibitor attenuated the irisin-induced HUVEC proliferation. HUVECs were pretreated with the ERK inhibitor U0126 for 30 min followed by irisin treatment. (A) Western blots analyzed phosphorylated and total ERK proteinexpression. (B) Densitometric analysis of the related bands was expressed as the relative optical band density, which was corrected using respective total proteins as a loading control and normalized against the untreated control. The data were expressed as the mean ± SE of three independent experiments,**p<0.01 vs. untreated group. The effect of the ERK inhibitor on irisin-induced HUVECproliferation was analyzed by Ki67 staining (C, D) and [^3^H] thymidine uptake (E). The data were expressed as the mean ± SE of three independent experiments, **p<0.01 vs. the irisin-treated group.

To further examine that irsin enhances HUVEC proliferation through the ERK signaling pathway, U0126 (ERK inhibitor) was used to block ERK activation. Western blot results demonstrated that the irisin-induced ERK phosphorylation was significantly suppressed, while there was no reduction in the amount of total ERK protein (Fig. 4A and B). The irisin-stimulated HUVEC proliferation was evaluated by measuring Ki67 staining in each group (Fig. 4C and D) and [^3^H] thymidine uptake (Fig. 4E). The data demonstrated that the r-irsin-induced increase of [^3^H] thymidine uptake was significantly reduced by U0126 treatment. Similar results were observed using immunofluorescent staining, which also demonstrated that blocking ERK activation mitigated irisin-mediated proliferation.

### Irisin protects HUVECs from high glucose-induced apoptosis

**Figure 5:**
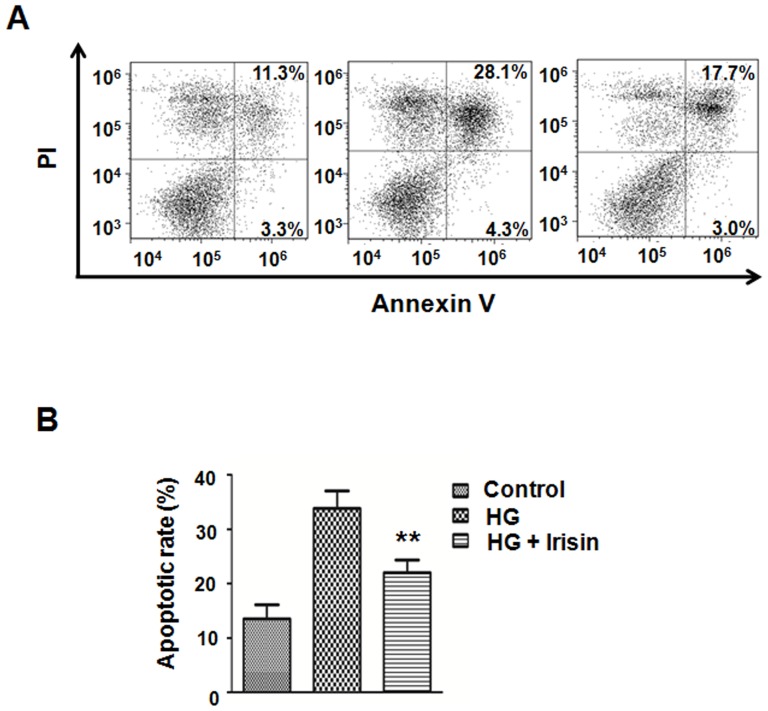
Irisin rescued HUVECs from high glucose-induced apoptosis. (A) Annexin V binding and propidium iodide (PI) staining was analyzed by FlowJo. (B)The bar graph represents the results of three independent experiments, **p<0.01 vs. the high glucose-treated group.

To determine whether irisin has a direct effect on HUVEC apoptosis, the cells were incubated with high glucose and irisin for 24 hours. As demonstrated in the flow cytometry results, irisin effectively attenuated high glucose-induced apoptosis in HUVECs at a dose of 20 nM (Fig. 5A and B). The percentage of cells undergoing apoptotic cell death decreased from 33.8±3.2% in the high glucose group to 22.0±2.4% after being exposed to 20 nM irisin for 24 hours.

### Irisin down-regulates Bax, Caspase-9, Caspase-3 and up-regulates Bcl-2 expression in HUVECs on high glucose conditions

**Figure 6:**
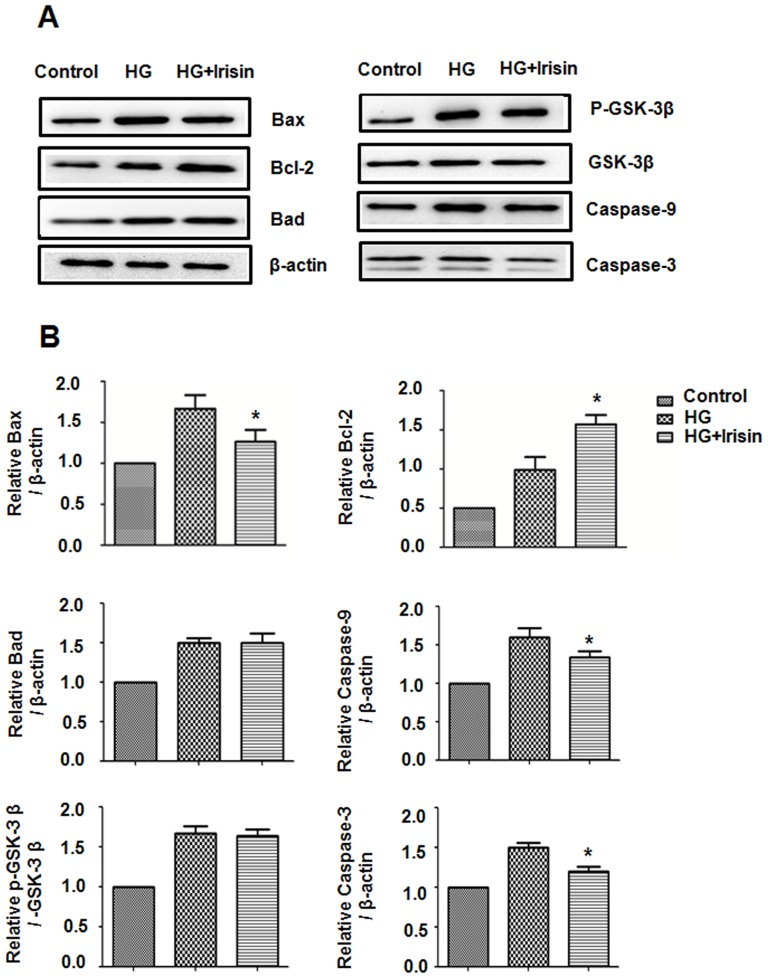
Irisin mediates Bax,Bcl-2,GSK-3β, Caspase-9 and Caspase-3 protein levels in HUVECs. HUVECs were cultured with or without irisin (20 nM) for 24 h. Bax, Bcl-2, Bad, GSK-3β, Caspase-9 and Caspase-3 in cell lysates were analyzed by Western blot. (B) Densitometric analysis of the related bands was expressed as the relative optical band density and was corrected using respective total proteins as loading controls and normalized against the untreated control. The data were expressed as the mean ± SE of three independent experiments, *p<0.05 vs. the high glucose-treated group.

To further investigate the potential mechanism of irisin on high glucose-induced HUVEC apoptosis, the impact of irisin on expression of Bcl-2, Bax, Bad, GSK-3β,Caspase-9 and Caspase-3, several key apoptosis regulator proteins, was examined [12], [13]. The Western blot results indicated that following the treatment with 20 nM irisin, expression of protein Bax, Caspase-9 and Caspase-3 decreased and the anti-apoptotic protein Bcl-2 increased. The expression of GSK-3β and Bad were not influenced. (Fig. 6A and B).

## Discussion

Irisin, which was discovered in 2012 by Bostrom and colleagues, is a cleaved and secreted fragment of FNDC5. Irisin is released by skeletal muscle and is increased with exercise, can act on different tissues and functions as a muscle-derived energy expenditure signal by promoting brown adipocyte thermogenesis in WAT both *invitro* and *in vivo* [14]. Furthermore, a recent study in the hippocampus demonstrated that endurance exercise increased the expression of irisin which lead to a significant increase in BDNF gene expression [15]. And another study showed the potential role of irisin on bone metabolism via modulating osteoblast differentiation [16]. Over-expression of irisin by intravenous injection of adenoviral vectors significantly increased total body energy expenditure, reduced body weight, and improved metabolic parameters such as insulin sensitivity in mice that were fed a high fat diet [5]. Moreover, serum irisin levels are lower in type 2 diabetes mellitus patients than in controls with normal glucose tolerance [6], [7]. Irisin concentrations in breast milk and plasma are also lower in lactating women with gestational diabetes mellitus than in non-lactating or healthy lactating women [17]. Because of these data, irisin has been proposed as a meaningful therapeutic target for diseases caused by inactivity or chronic caloric excess such as diabetes and obesity. Meanwhile, some controversial results and conclusions against the beneficial roles about irisin have recently been reported [18]–[20]. Among of these reports, a study found a positive correlation between circulating irisin levels and body weight, BMI, fat mass [18]. But another study have drawn an opposite conclusion [19]. The discrepant results raise serious concerns about its application prospect. So further research need to be made.

Diabetes and hyperglycemia are intimately involved with endothelial dysfunction and markedly increase the risk of all forms of cardiovascular complications including critical limb ischemia and foot ulcers [21], [22]. However, besides an interaction between physical activity and metabolic homeostasis, no previous studies have evaluated whether irisin may directly regulate endothelial cell proliferation and apoptosis. Recently, our laboratory successfully established an efficient system for the expression and purification of human recombinant irisin protein in *Pichiapastoris* [10]. In the current study, it was demonstrated that irisin can effectively promote HUVEC proliferation by activating the ERK signaling pathway, while p38 MAPK and Akt appeared to be uninvolved. Moreover, in addition to serving as a potent proliferation factor, our data revealed that irisin also inhibited high glucose-induced HUVEC apoptosis. Because regulating endothelial cell proliferation and apoptosis can significantly affect vascular endothelial integrity and sustain endothelium homeostasis [3], the effect of irisin on regulating HUVEC proliferation and apoptosis of may elucidate both the prevention and treatment of various vascular diseases, especially for some metabolism-related vascular diseases, because of its potential therapeutic role in metabolism.

It is widely believed that ERK and PI3K/Akt signaling promotes cell proliferation by affecting DNA synthesis [23], [24]. Our previous study reported that the ERK pathway can be activated by irisin to modulate the irisin-induced emergence of brown adipocytes. In this study, our data demonstrate that irisin significantly increased ERK phosphorylation, while the levels of P-p38 and P-Akt were not different. Furthermore, because treatment with the ERK inhibitor U0126 inhibited cell proliferation, it was concluded that irisin-induced HUVEC proliferation is dependent on ERK signaling pathway activation. However, the pro-proliferative effect of irisin in HUVECs was only partially abolished, implying that there were alternative signaling pathways contributing to irisin-induced HUVEC proliferation.

In general, apoptosis is regulated by pro-apoptotic and anti-apoptotic proteins and is executed through caspases [25]. Bcl-2 protein family members play an essential role in regulating and executing many cell apoptotic pathways. Examples include Bcl-2, Bax and Bad, which are members of the Bcl-2 family that function as inhibitors and activators of apoptosis, respectively [12], [13]. The present study demonstrated that irisin attenuated high glucose-induced HUVEC apoptosis. The flow cytometry result was accompanied by increased Bcl-2 expression and decreased Bax expression. It is well known that Akt is a potent mediator of cell proliferation and cell survival. Previous study demonstrated that Akt promoted cell survival and block apoptosis via its downstream targets, including GSK-3, Bad, caspase-9 [26]. Akt inhibits the conformational change and translocation to mitochondria of Bax protein, so as to prevent the caspase-3 activation [27]. The present study found that irisin did not affect the expression of GSK-3β and Bad, the downstream targets of Akt. But the expression of caspase-9 and caspase-3 were downregulated when treated with irisin. These results imply that irisin may mediated the HUVEC survival via Akt-independent pathway and its precise mechanism need to be further study.

## Conclusions

We presented data demonstrating that recombinant irisin promoted HUVEC proliferation and activated the ERK signaling pathway. Irisin also protected cells from high glucose-induced apoptosis by up-regulating Bcl-2 levels together with down-regulating Bax,Caspase levels. Although we studied several signaling pathways that are primary targets of cell proliferation and apoptosis, there could still be other signaling pathways that are involved. Because the actions of irisin may be different between *in vitro* and *in vivo*, future work is needed to determine the physiological effects of irisin in mice and humans.
